# Machine Learning Prediction of the Compressive Bearing Capacity of Concrete-Filled Steel Tubes Using Random Forest

**DOI:** 10.3390/ma19122511

**Published:** 2026-06-10

**Authors:** Weidi Su, Yaofei Cheng, Li Wei, Guangda Zhong, Linxiao Zhou, Fei Liu, Kaizhong Xie

**Affiliations:** 1 Guangxi Pinglu Canal Construction Co., Ltd., Nanning 530004, China; suweidi@plyh.gx.cn (W.S.); chengyaofei@plyh.gx.cn (Y.C.); zhongguangda@plyh.gx.cn (G.Z.); 2 Guangxi Road Construction Engineering Group Co., Ltd., Nanning 530004, China; 13687711427@163.com; 3 Guangxi Laboratory of Modern Canal, Nanning 530004, China; liufei648@163.com (F.L.); xiekaizhong@163.com (K.X.); 4 College of Civil Engineering and Architecture, Guangxi University, Nanning 530004, China

**Keywords:** concrete-filled steel tube (CFST), compression member, random forest model, bearing capacity prediction

## Abstract

**Highlights:**

- High-precision RF model for CFST capacity using 154 tests and 24 inputs.
- Steel tube inertia (*I_s_*) dominates prediction (35.56% contribution).
- Validated by nine CFST tests with prediction errors within 5%.

**Abstract:**

Concrete-filled steel tube (CFST) members are widely used in long-span and high-rise structures due to their high load-bearing capacity and structural efficiency. Accurate prediction of their compressive bearing capacity is essential for reliable design. In this study, a data-driven prediction model based on the Random Forest (RF) algorithm was developed using a database of 154 axial compression tests. A total of 24 parameters, including geometric dimensions, material properties, and sectional characteristics, were considered as input variables, and the model was optimized through five-fold cross-validation and hyperparameter tuning. The results indicate that the proposed model achieves high accuracy and stability, with mean predicted-to-experimental ratios of 1.002 and 0.989 for the training and testing sets, respectively, and maximum deviations within 15%. Compared with existing design codes and alternative machine learning methods, the RF model improves prediction accuracy by approximately 9% and exhibits strong generalization capability. Furthermore, independent experimental validation using nine CFST column tests confirms its reliability, with prediction errors within 5%. These findings demonstrate that the proposed model provides an effective and practical tool for predicting the compressive bearing capacity of CFST members in engineering applications.

## 1. Introduction

**Figure 1:**
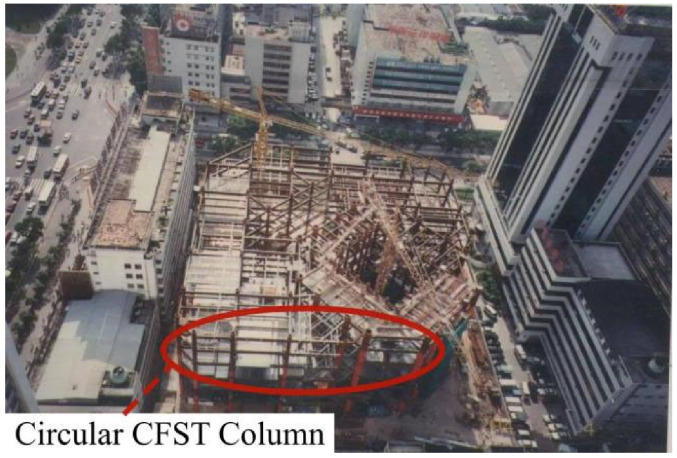
Application of CFST in practical engineering.

**Figure 2:**
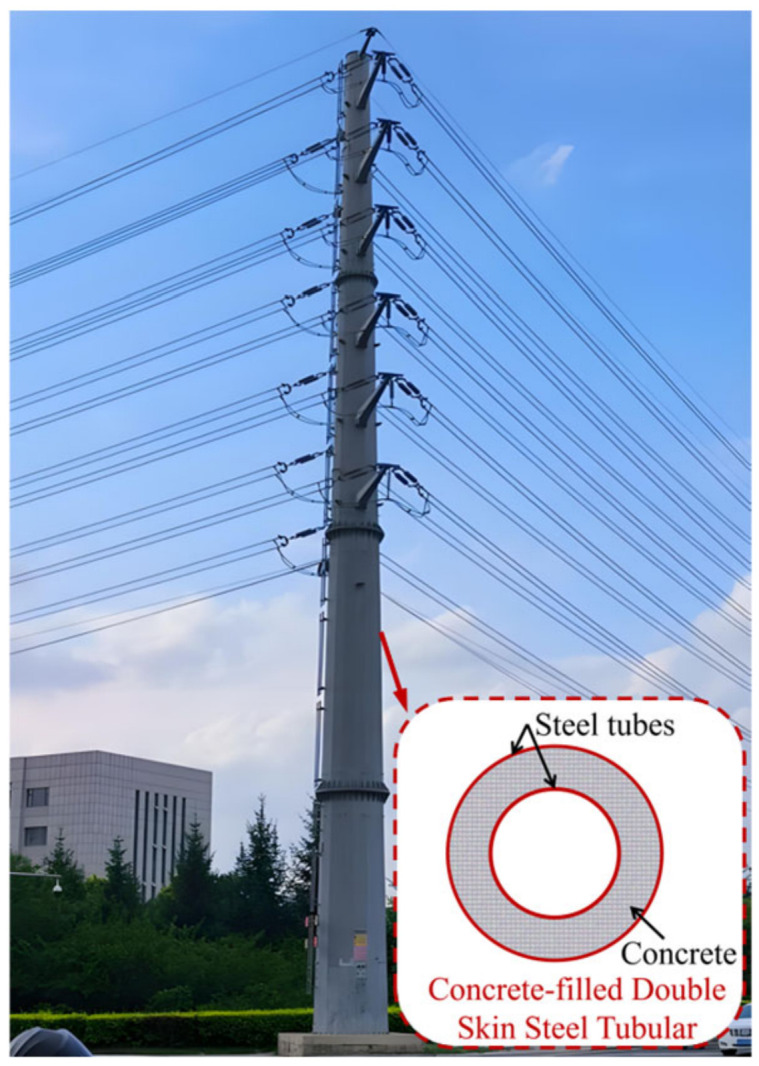
Application of CFDST structures in power transmission engineering.

Steel-concrete composite structures have been extensively applied in engineering fields such as bridges, high-rise buildings, and large-span spatial structures, owing to their outstanding advantages, including high bearing capacity, large stiffness, excellent seismic performance, and significant economic benefits [1]. To illustrate these structural advantages in practice, Figure 1 and Figure 2 present typical engineering applications where CFST members serve as critical load-bearing components. Specifically, Figure 1 shows the application of CFST in practical engineering, while Figure 2 illustrates concrete-filled double skin steel tubular (CFDST) structures in power transmission engineering. Representative examples also include super high-rise buildings like the SEG Plaza and CITIC Plaza [2,3,4]. Furthermore, these structures are widely utilized in large-scale infrastructure projects, such as the Pinglu Canal housing construction, which adopts a “permanent–temporary integrated” construction mode, wherein CFST-based composite structural members are extensively employed as critical load-bearing components. This practice provides a replicable engineering paradigm for the broader application of steel-concrete composite structures in major infrastructure projects [5]. However, as complex hybrid systems, the mechanical behaviors of structures in steel-concrete composite structures, such as concrete-filled double skin steel tubular (CFDST) structures, as shown in Figure 2, exhibit mechanical behaviors influenced by the coupling effects of various complex factors, including material nonlinearity, geometric nonlinearity, interface slip, construction errors, and initial imperfections [2,6]. Accurately predicting the ultimate bearing capacity of members under various loading conditions has become a core challenge in structural design and safety assessment [7,8,9]. Traditional calculation methods based on superposition theory ignore the complex interactions between steel and concrete, resulting in limited computational accuracy. In particular, when dealing with high-strength materials, complex stress states, or novel section types, these methods face severe challenges in terms of applicability and accuracy [10].

Researchers studying the ultimate bearing capacity of traditional steel-concrete composite structures typically employ unified theory and superposition theory as theoretical models for calculating ultimate bearing capacity [11]. Unified theory treats the CFST components in steel-concrete composite structures as an integrated unit, solving for ultimate load through equilibrium differential equations that consider material nonlinearity and geometric imperfections. While it can better reflect the interactions between materials, its mathematical derivation is complex and requires numerous simplifying assumptions, limiting its application under complex loading conditions [12,13]. In contrast, superposition theory is simpler and more practical, treating bearing capacity as the simple superposition of the bearing capacities of steel and concrete, and roughly considering confinement effects through confinement coefficients [14]. While this theory facilitates engineering design, it cannot be applied to torsional members and fails to account for the complex interactions between steel and concrete, presenting deficiencies in calculating ultimate axial compressive strength [15].

To meet the demands of modern engineering, researchers have begun exploring the engineering applications of machine learning algorithms. While classical algorithms like support vector machines excel at handling small-sample and nonlinear problems but are sensitive to hyperparameters and exhibit lower training efficiency [16,17]. In contrast, recent advancements in Deep Learning (DL) and artificial neural networks possess powerful function approximation capabilities; they heavily rely on massive datasets and suffer from poor interpretability, making them less ideal for structural engineering, where experimental data is often limited and physical interpretability is mandatory [18]. For the complex regression problem of CFST ultimate bearing capacity, which involves high nonlinearity and parameter coupling, ensemble learning methods demonstrate significant advantages. Although gradient boosting decision trees slightly outperform Random Forests in accuracy, they require longer training times and are more sensitive to outliers [19,20]. Conversely, the Random Forest (RF) algorithm reduces the risk of over-parameterization through ensemble learning, provides robust generalization on small-to-medium datasets, and uniquely offers feature importance evaluation, which is crucial for engineering validation and physical interpretability. Meanwhile, due to parallel training characteristics and robustness to noise, Random Forests demonstrate superior performance in computational efficiency and model stability.

As an ensemble learning algorithm, Random Forest reduces the risk of over-parameterization by constructing multiple decision trees, featuring high robustness and generalization capability, insensitivity to noise, and the ability to evaluate feature importance [21,22]. It has been widely applied in fields such as ground surface settlement prediction and slope stability analysis [23,24,25,26]. For the complex regression problem of ultimate bearing capacity of steel-concrete composite structural members, which is highly nonlinear and involves parameter coupling [27], Random Forest demonstrates natural applicability, enabling high-precision prediction for axially and eccentrically compressed members [28,29]. Meanwhile, the field of steel-concrete composite structures has accumulated abundant experimental and numerical simulation data, providing a solid foundation for training high-precision models [21,30,31,32]. In summary, with high precision, high efficiency, strong robustness, certain interpretability, and extensive data support, Random Forest has become an ideal tool for solving the complex nonlinear regression problem of ultimate bearing capacity prediction for CFST members.

A Random Forest-based machine learning predictive model is proposed in this study to estimate the ultimate bearing capacity of CFST members. To validate the model, a series of axial compression tests was performed on CFST specimens, involving comprehensive characterization of the material properties of steel tubes and core concrete, as well as measurement of the ultimate compressive capacities. The results indicate that the RF model yields predictions in close agreement with the experimental data, thereby verifying both its computational accuracy and practical applicability. Ultimately, the novelty of this study lies in explicitly quantifying the structural mechanics hierarchy through data-driven feature importance, revealing that the steel tube inertia dominates the prediction with a 35.56% contribution. Unlike existing black-box ML models that focus solely on prediction accuracy, this quantification provides new physical insights that complement classical CFST theory, demonstrating that the top seven parameters collectively account for over 80% of the variance, which aligns with the confinement mechanism. Furthermore, the proposed model achieves a mean predicted-to-experimental ratio of 0.989 for the testing set with maximum deviations within 15%, improving prediction accuracy by approximately 9% compared with existing design codes, and is rigorously validated by nine independent CFST column tests with prediction errors within 5%.

## 2. Experimental Program

### 2.1. Experimental Database

To accurately predict the ultimate bearing capacity of steel-concrete components using a Random Forest (RF) model, a comprehensive dataset of axially compressed specimens is essential. This study compiled 154 experimental datasets from the literature [33,34,35,36,37,38,39,40,41,42,43], encompassing a wide range of material strengths and slenderness ratios: 18.0 ≤ *D*/*t* ≤ 165, 8.4 ≤ *λ* ≤ 168, 216 ≤ *f_y_* ≤ 617.8, and 16 ≤ *f_ck_* ≤ 121.1, ensuring good sampling comprehensiveness. The first 24 parameters, including structural dimensions such as the diameter (*D*) and wall thickness (*t*) of the concrete-filled steel tube and the column length (*L*), serve as the input data. The 25th parameter, bearing capacity (*N_t_*), is the sole output for prediction. Descriptive statistics were computed for the entire dataset, including count, mean, standard deviation (std), minimum (min), 25th percentile (25%), median (50%), 75th percentile (75%), and maximum values (max), as shown in Table 1.

The dataset, comprising 154 samples, provides a sufficient basis for model training and validation, as it comprehensively encompasses the critical parameter space, and data characteristics were further analyzed using variable boxplots and violin plots.

**Figure 3:**
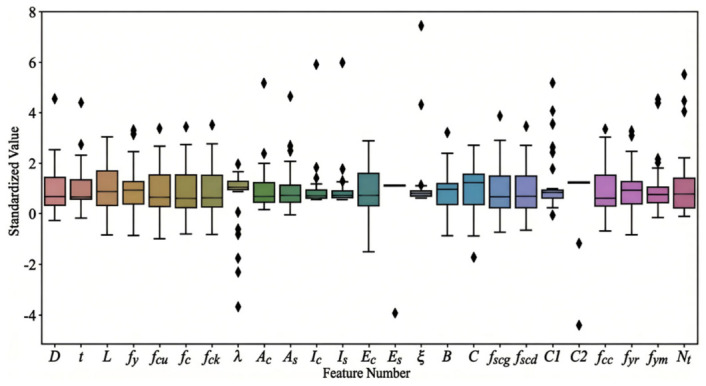
Standardized data boxplot.

Based on the component model variables, we conducted a detailed analysis of the overall distribution of the data. After incorporating the feature data, we plotted a boxplot, as illustrated in Figure 3.

**Figure 4:**
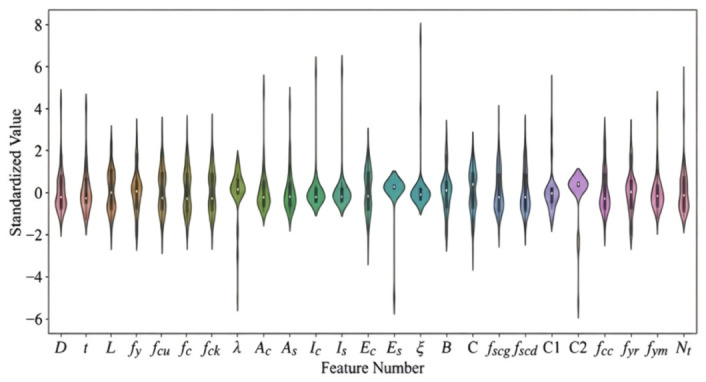
Standardized data violin plot.

The boxplot indicates that features *D*, *t*, *L*, *f_y_*, *f_cu_*, *f_c_* and *f_ck_* are symmetrically distributed around the mean, with upper and lower quartiles within [−2.5, 2.5]. Features *λ*, *ξ*, and *C*_1_ show pronounced outliers, with *λ* mainly below the mean and *ξ* and *C*_1_ primarily above it. Features *l_c_*, *l_s_*, *E_s_*, *B* and *f_ym_* cluster around the mean, exhibiting minimal variance. Due to the large number of variables, violin plots were employed to present the distributions more clearly and concisely, as shown in Figure 4.

The violin plot indicates that features *L*, *f_cu_*, *f_c_*, *f_ck_*, *E_c_* and *C* exhibit bimodal distributions, while *t*, *f_y_*, *λ*, *A_c_*, *A_s_*, and *f_my_* display unimodal distributions. Features *A_c_*, *A_s_*, *l_c_*, *l_s_*, and *ξ* show long upper tails, whereas *λ* and *E_s_* have long lower tails. These long-tailed patterns represent valid outliers arising from natural variability in data generation rather than measurement errors, and therefore do not compromise the integrity or suitability of the dataset for analysis.

### 2.2. Data Quality and Heterogeneity

The 154 groups of experimental data sources compiled in this paper provide rich sample diversity for model training. However, multi-source data fusion inevitably introduces heterogeneity, so it is necessary to carefully evaluate the data quality. In terms of data consistency, different studies may have differences in the loading system, end constraint conditions and measurement methods of specimen geometry. The descriptive statistical results of some variables in Table 1 and the outliers presented by *λ*, *ξ* and *C*_1_ in Figure 3 and Figure 4 not only reflect the natural variability of the parameters themselves, but also may be related to the systematic differences in test conditions between studies. This study retains these outliers for analysis, on the one hand, because they conform to the real physical mechanism of data generation, on the other hand, eliminating outliers may lead to the prediction distortion of the model in the extreme parameter range.

In terms of measurement uncertainty, the determination of steel yield strength *f_y_* depends on the tensile test equipment and sample processing accuracy in different laboratories. The compressive strength of concrete cube *f_cu_* is affected by factors such as curing conditions, loading rate and the flatness of the end face of the test block, and the point selection method of *E_c_* may also be inconsistent among different sources. In view of the fact that most of the source documents do not completely report the above measurement details, this paper has uniformly adopted the measured mean value of each document report in the process of data collection, and assumed that the measurement errors of each source were randomly distributed within the acceptable engineering tolerance.

## 3. Research Methodology

### 3.1. Basic Principles of Random Forest Method

RF utilizes ensemble learning, consisting of multiple classification and regression trees (CARTs). A decision tree is a statistical model that outputs different classes or values based on input features [44]. Results are obtained by randomly selecting features for each tree, and then majority voting or averaging is applied based on the specific problem. The final prediction is a stable and accurate result, as shown in Equation (1). Given input data {*H (x*, *θ_i_)*, *i =* 1, 2, …, *k*}, the Random Forest prediction is the average of all individual decision tree predictions {*H* (*x*, *θ_i_*)}.

(1) $$\bar{H}\left(x\right)=\frac{1}{k}\sum_{i=1}^{k}H\left(x,\theta_{i}\right)$$

In the formula, *H* (*x*) represents the predicted value of the Random Forest model, *θ_i_* denotes the random variable of a single decision tree, *x* is the feature variable, and *k* is the number of decision trees. The Random Forest algorithm uses bootstrapping, randomly sampling *k* times from the original data repository, with each decision tree trained on a sample of the same size as the original. Each sample set includes data from the out-of-bag (OOB) data, as shown in Equation (2). The *q* training sample sets are drawn using Bootstrap sampling to construct *q* decision trees. The input sample set is

(2) $$D=\left\{\left(x_{1},y_{1}\right),\left(x_{2},y_{2}\right),\ldots \ldots ,\left(x_{N},y_{N}\right)\right\}$$

Among them, the weak classifier has a certain number of iterations, and the final strong classifier *H* (*x*) is the output.

### 3.2. Model Development

Random Forest (RF) comprises multiple independent decision trees, each structured with nodes, branches, and leaf nodes, where nodes test specific attributes to split the tree. Individual decision trees are highly sensitive to training data and prone to over-parametrization [44]. The RF algorithm mitigates this issue through bagging [45], enhancing robustness. In this study, the number of trees was set between 10 and 200, with 5-fold cross-validation applied. Key parameters—maximum number of trees, maximum depth, minimum samples per leaf, minimum samples per split, and maximum features—were optimized based on their effects on mean squared error (*MSE*) to achieve optimal performance. It is possible that additional validation methods could also be worth considering. That said, the approaches currently adopted might perhaps be regarded as providing a relatively thorough evaluation of predictive accuracy.

#### 3.2.1. 5-Fold Cross-Validation Results

Following K-fold cross-validation principles for Random Forest [46], modeling was conducted using 5-fold cross-validation. The dataset was split into 80% for training and 20% for testing. The component database underwent preprocessing prior to modeling and analysis, with the procedure outlined as follows:

(1) Input the following sample set:

(3) $$D=\left\{\left(x_{1},y_{1}\right),\left(x_{2},y_{2}\right),\ldots \ldots ,\left(x_{N},y_{N}\right)\right\},N=123$$

Among them, the number of iterations for the weak classifier *T* = 25, and the final strong classifier *H* (*x*) is output.

(2) For T = 1, 2, …, 25, the training set undergoes the *T*-th bootstrap sampling, repeated 123 times to generate a sampling set DT of 123 samples. The model comprises 25 decision trees. For instance, in constructing the 19th tree, 123 bootstrap iterations on the 123 training samples yield 79 unique samples, while m features are randomly selected for splitting. These 79 samples serve as the training set for model computation and output generation:

(4) $$x^{j}=A_{s},s=-0.223$$

Feature *A_s_* is chosen as the root node, splitting the binary tree into left and right subtrees at −0.223. These values are then used to determine the optimal split boundary *j* and split point *s*, producing the current output.

(5)  value=7.272,squared_error=0.427

The 79 sample points are evaluated against the condition *A_s_* ≤ −0.223. An amount of 38 points satisfies the condition, while 41 do not. The 38 satisfying points are used in the calculation to obtain *x^j^* = *A_c_* and *s* = −0.756, which is then used to compute the current output value.

(6)  value=6.752,squared_error=0.231

The 38 sample points are evaluated against *A_s_* ≤ −0.756, resulting in 6 valid and 32 invalid samples. The process is repeated for the 6 valid points to determine *x^j^* = *f_scg_* and *s* = −0.882, which is then used to compute the current output value.

(7)  value=5.894,squared_error=0.003

The 6 sample points are evaluated against *f_scg_* ≤ −0.882, with 4 satisfying the condition and 2 not. The procedure is repeated for the 4 satisfying points to determine *x^j^* = *ξ* and *s* = −0.255, which are then applied to compute the current output value.

(8)  value=5.858,squared_error=0

**Figure 5:**
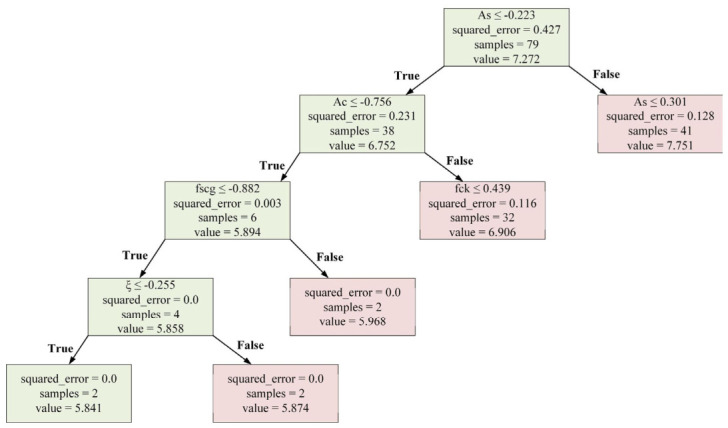
The complete 19th decision tree.

The stopping condition is met by evaluating the four sample points against *ξ* ≤ −0.255, with two nodes satisfying the condition, producing an output of 5.841 and forming a leaf node. Recursively returning to the previous node, the remaining two nodes are false, yielding an output of 5.874 with zero squared error, forming another leaf node. Similarly, two samples with *f_scg_* ≤ −0.882 are false, resulting in an output of 5.968 and a squared error of 0, creating an additional leaf node. Continuing this recursive process constructs the complete 19th decision tree, 19D, as shown in Figure 5.

The decision tree construction is repeated 25 times, generating 25 trees. Bagging is applied, and the regression outputs are averaged to form the final Random Forest prediction model. The predictions are evaluated using appropriate metrics on 123 samples.

(9) $$MSE=\left(\sum_{L}^{i=1}{\left(y^{obs}-y^{\mathrm{pred}}\right)}^{2}\right)/L$$

The calculation yields *MSE* = 0.0081; as further validation, calculating R^2^ and MAE ensures the accuracy of the model and adds more robust evaluation metrics.

(10) $$R^{2}=1-\frac{SS^{res}}{SS_{\mathrm{tot}}}\;\;\;$$

The calculation result shows that R^2^ ≈ 0.9791.

(11) $$\frac{1}{n}\sum |y_{i}-{\hat{y}}_{i}|\;\;$$

The calculation result shows that MAE = 112.33. Based on the range of true values (348~5927), the proportion of MAE is relatively small, consistent with R^2^ = 0.979 and MSE = 0.0081, indicating that the model has high accuracy.

##### 3.2.2. Parameter Combination Optimization

The decision tree parameters—max decision trees, max depth, min sample leaf, min samples split, and max features—along with the training set division ratio, are optimized to improve the accuracy of the RF prediction model. These parameters are combined and fine-tuned to achieve the best prediction performance.

**Figure 6:**
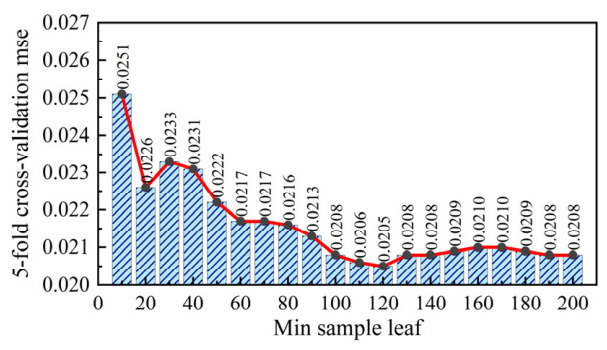
Decision tree quantity error.

**Figure 7:**
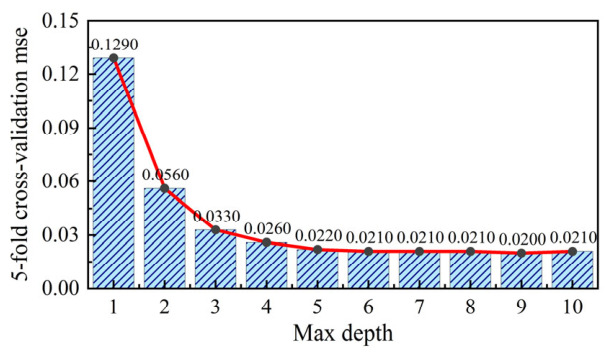
Relationship between max depth and error.

**Figure 8:**
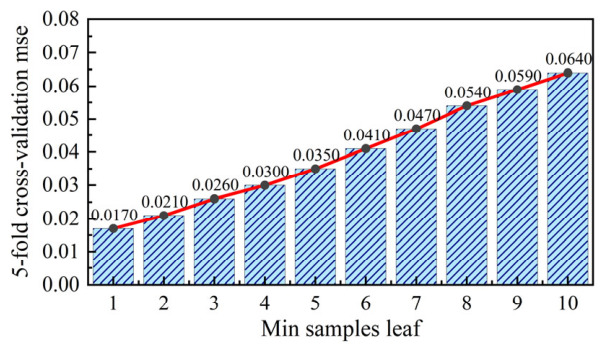
Relationship between min sample leaf and error.

**Figure 9:**
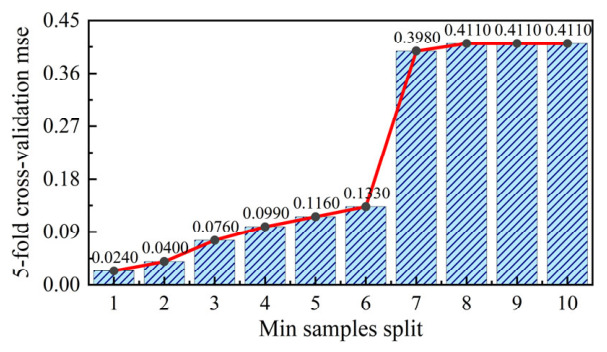
Relationship between min samples split and error.

**Figure 10:**
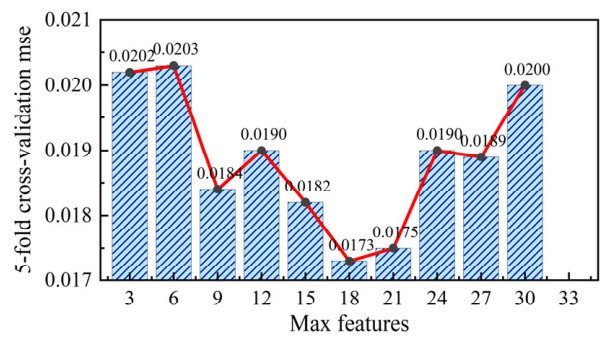
Relationship between max feature and error.

**Figure 11:**
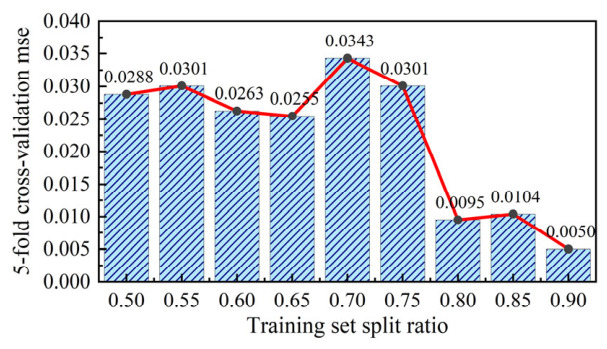
Relationship between training set split ratio and error.

The optimization results indicate that the number of decision trees stabilizes at an *MSE* of 120, with 25 trees selected for optimal accuracy and efficiency, as shown in Figure 6. The maximum subtree depth achieves stable *MSE* beyond a depth of 5, and a depth of 10 prevents over-parametrization while maintaining accuracy, as shown in Figure 7. The minimum leaf samples are optimal at 2, as shown in Figure 8, and the minimum split samples perform best at the default value of 2, as shown in Figure 9. The maximum number of features is optimal at 10, as shown in Figure 10, ensuring low *MSE* and model stability. An 80% training set proportion provides low *MSE* and reliable results, as shown in Figure 11.

The optimization results indicate that the number of decision trees stabilizes at an MSE of 120, with 25 trees selected for optimal accuracy and efficiency. Maximum subtree depth stabilizes after 5, and a depth of 10 prevents over-parametrization while maintaining accuracy. The minimum leaf samples are optimal at 2, and the minimum split samples perform best at the default of 2. The maximum number of features is optimal at 10, ensuring low *MSE* and stability. An 80% training set proportion produces low *MSE* and reliable results.

## 4. Model Prediction Results

### 4.1. Result Coefficient

**Figure 12:**
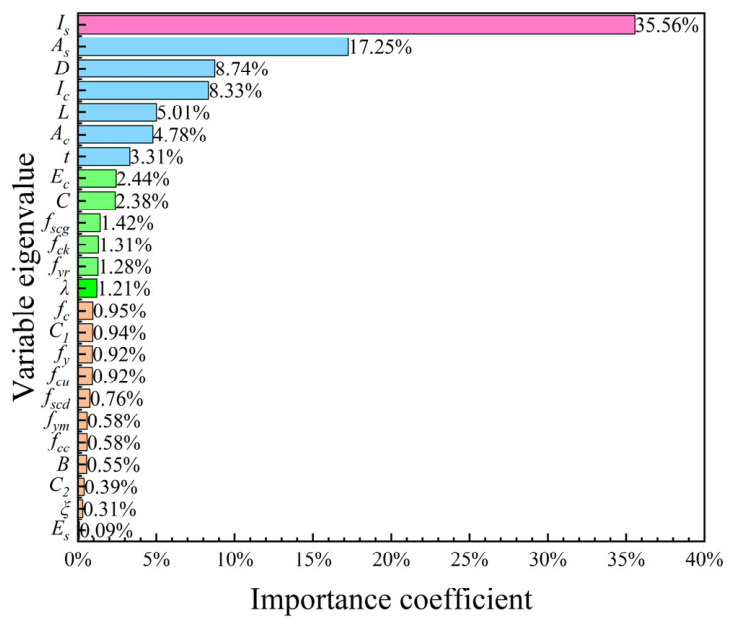
Model characteristic importance coefficient.

The importance coefficients from the optimized parameter model in Section 3.2.2 are shown in Figure 12. The contribution of each independent variable to the RF model is based on the largest or relatively significant coefficients. Among the 24 features, *I_s_* contributes the most, accounting for 35.56%. *A_s_*, *D*, *I_c_*, *L*, *A_c_*, and *t* account for 17.25%, 8.74%, 8.33%, 5.01%, 4.78%, and 3.31%, respectively. *E_c_*, *C*, *f_scg_*, *f_ck_*, *f_yr_*, and *λ* contribute between 1.21% and 2.44%, with respective contributions of 2.44%, 2.38%, 1.42%, 1.31%, 1.28%, and 1.21%. Other variables contribute less than 1%. This shows that the ultimate bearing capacity is primarily influenced by *I_s_*, with factors like *A_s_*, *D*, *I_c_*, *L*, *A_c_*, and *t* also playing important roles. In practical engineering, careful attention should be given to selecting values for these variables. Although the impact of variables like *E_c_*, *C*, *f_scg_*, *f_ck_*, *f_yr_*, and *λ* is smaller, they should be adjusted with appropriate reduction factors when constructing the bearing capacity equation for comprehensive analysis.

While the Random Forest model assigns feature importance values (e.g., *I_s_* = 35.56%), it is essential to link these results to structural mechanics principles to provide engineering insight. For instance, the high contribution of the steel ratio (*I_s_*) aligns with classical CFST column theory, where the steel content significantly affects both confinement and ductility. Higher steel ratios improve the column’s ability to resist axial loads and enhance post-yield behavior due to the interaction between steel and concrete.

Similarly, the concrete compressive strength, which also shows notable importance, directly influences the ultimate axial capacity. According to the standard design formula, the axial strength of CFST columns is a function of both steel and concrete contributions. The model’s feature importance reflects this underlying physical relationship, validating that the ML predictions are not only statistically accurate but also physically meaningful.

This interpretability enables engineers to understand which parameters most strongly affect performance, supporting informed design and optimization of CFST structures, beyond the purely predictive capability of the model.

**Figure 13:**
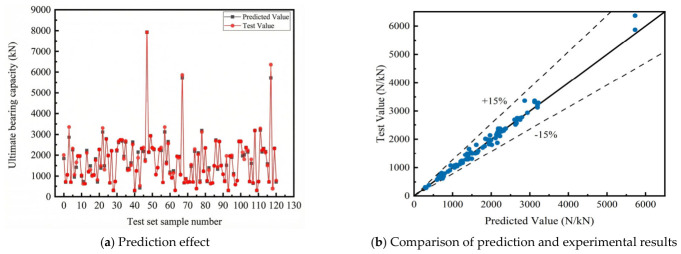
Training set prediction of Random Forest model.

**Figure 14:**
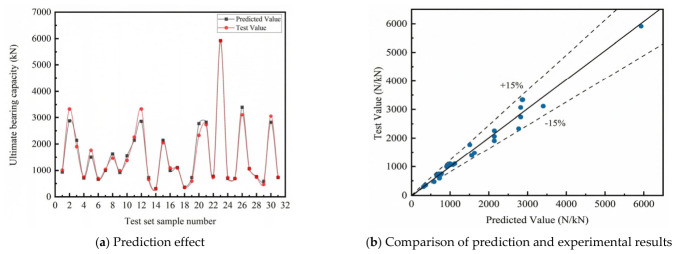
Test set prediction of Random Forest model.

Optimized training samples from 154 datasets capture the macroscopic mechanical behavior of component ultimate bearing capacity under various conditions. Statistical results are presented in Table 2, enabling a clear comparison between predicted and experimental values. As shown in Figure 13a and Figure 14a, the RF model predictions for the training (80%) and test (20%) sets closely align with experimental results, with curves nearly overlapping, indicating minimal deviation and high accuracy. Figure 13b and Figure 14b show that predicted points are evenly distributed, with most coinciding with the isoline and maximum deviations within 15%, confirming strong generalization and avoidance of overfitting. These results validate the improved RF model’s predictive reliability, supporting rapid and accurate estimation of ultimate bearing capacity for concrete-filled steel tubes.

Table 3 presents the average value (*AVG*), mean square deviation (*σ*), and coefficient of variation (*COV*) for the ratio between predicted and experimental values. The low σ and COV indicate minimal deviation between predicted and actual values.

**Figure 15:**
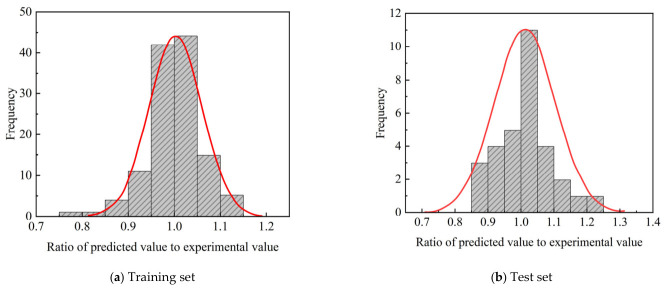
Ratio distribution of ultimate bearing capacity between member prediction and test.

Figure 15a,b show the histogram distributions of predicted-to-experimental ratios of ultimate bearing capacity for the training and testing datasets. For both datasets, the median and mean of the ratio distributions are close (1.0008 and 1.0020 for training, 0.9849 and 0.9890 for testing), indicating minimal bias and high accuracy of the improved RF prediction model.

For practical engineering applications, the proposed RF model can be deployed as an interactive computational tool or Application Programming Interface (API), enabling the direct input of the 24 parameters for rapid bearing capacity estimation without requiring machine learning expertise. Based on the feature importance analysis, priority should be given to the selection of *I_s_*, *A_s_* and *D* during the preliminary design phase, as these parameters dominate the prediction. However, the practical application of this model has inherent limitations: Predictions are reliable only within the parameter ranges of the training dataset; extrapolation beyond these bounds may lead to inaccuracies. The current model does not explicitly account for long-term loading effects, local buckling, or initial geometric imperfections beyond their implicit capture in the experimental dataset. Future work should incorporate numerical simulation data to expand the applicable parameter space and physical constraints.

### 4.2. Discussion on Physical Interpretability

The RF is a typical data-driven model, and the order of characteristic importance revealed by it is highly consistent with the classical mechanical behavior of CFST, which provides a strong physical basis for the prediction results of the model.

The results showed that *I_s_* (35.56%), *I_c_* (8.33%), *D* (8.74%) and *L* (5.01%) were the most important characteristics. These parameters jointly define the slenderness ratio and section bending stiffness. In structural mechanics, slenderness ratio and flexural stiffness are the core parameters to control whether the overall buckling instability of members occurs under axial load. Especially for long- and medium-length columns, the ultimate bearing capacity is far lower than the section strength, which is mainly determined by the stability performance. This shows that the ranking of characteristic importance of the Random Forest model is not a random statistical result, but an accurate mapping of the physical reality that, under axial compression, the contribution of geometric stiffness is greater than that of material strength, which conforms to the basic principles of structural mechanics.

## 5. Comparative Analysis of Model Effects

### 5.1. Compare with Standard Procedures of Various Countries

Based on the experimental database, the results of the ultimate bearing capacity *N_0_* of components in various national standard specifications are calculated and compared with the results of the RF prediction model.

The ultimate bearing capacity *N*_0_ of components in the GB 50936-2014 [47] is

(12) $$N_{0}=A_{sc}f_{sc}=(1.212+B\xi +C\xi^{2})f_{c}$$

(13) $$B=0.176f_{s}/213+0.974$$

(14) $$C=-0.104f_{c}/14.4+0.031$$

where *f_sc_* and *A_sc_* represent the design value of composite section strength and section area, respectively; *B* and *C* are coefficients; *ξ* is the confinement coefficient of the member; and *f_c_* is the design value of concrete compressive strength.

The DBJ/T13-51-2010 [48] has a similar expression format to the aforementioned standards, but adopts different compressive strengths for CFST sections:

(15) $$f_{sc}=(1.14+1.02\xi )f_{c}$$

The AISC [49] specification calculates the axial compressive strength capacity based on the width-to-thickness ratio in three scenarios:

(16) $$\begin{array}{cc} N_{0}=f_{y}A_{s}+0.95f_{c}^{\prime }A_{c} & \mathrm{if}\;\lambda \leq \lambda_{p}\\ N_{0}=P_{p}-[P_{p}-(f_{y}A_{s}+0.7f_{c}^{\prime }A_{c})](\lambda -\lambda_{p}{)}^{2}/{\left(\lambda_{r}-\lambda_{p}\right)}^{2} & \mathrm{if}\;\lambda_{p}<\lambda \leq \lambda_{r}\\ N_{0}=0.72f_{y}A_{s}/[(D/t)f_{y}/E_{s}){]}^{0.2}+0.7f_{c}^{\prime }A_{c} & \mathrm{if}\;\lambda >\lambda_{r} \end{array}$$

where *λ_p_* and *λ*_0_ represent the limit values of the generalized width-thickness ratio.

The European Committee for Standardization EC4 Design Code for Steel-Concrete Composite Structures [50]:

(17) $$\;N_{0}=\eta_{s}f_{y}A_{s}+(1+\eta_{c}\frac{t}{D}\frac{f_{y}}{f_{c}^{\prime }})f_{c}^{\prime }A_{c}$$

where *η_s_* and *η_c_* represent the calculated parameters, respectively; *f^′^_c_* denotes the compressive strength of concrete cylinders.

The AIJ Code of the Architectural Institute of Japan [51]:

(18) $$\;N_{0}=1.27A_{s}f_{s}+0.85f_{c}^{\prime }A_{c}$$

(19) $$\;f_{s}=\min(f_{y},0.7f_{u})$$

where *A_s_* and *A_c_* represent the areas of steel tube and concrete, respectively; *f^′^_c_* denotes the compressive strength of the concrete cylinder; *f_s_* stands for the standard value of steel strength; *f_y_* signifies the yield strength of steel; and *f_u_* indicates the ultimate tensile strength.

British Standards Committee BS5400 Bridge Design Code [52]:

(20) $$\;N_{0}=0.95A_{s}f_{y}^{\prime }+0.45A_{c}f_{cc}$$

where *f^′^_y_* and *f_cc_* represent the strength indices of the steel tube and concrete, respectively.

**Figure 16:**
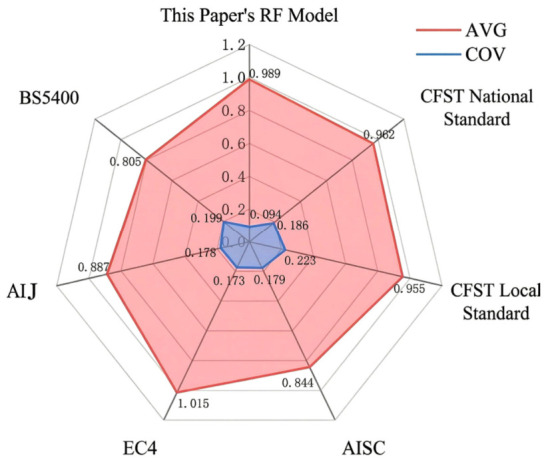
Comparison of calculation results of different standards for CFST members.

A comprehensive analysis of component ultimate bearing capacity considering data volume, calculation accuracy, and applicability of different standards is presented in Table 4 and Figure 16. Ratios of calculated results *N*_0_ from various standards to experimental results *N_t_*, along with mean values and coefficients of variation, are evaluated. Results indicate that AISC, AIJ, and BS5400 generally underestimate ultimate bearing capacity by over 10%, reflecting overly conservative predictions. In contrast, EC4 results tend to be on the unsafe side, with the landmark exhibiting the poorest stability. Using these two calculation methods produces unreasonable results. In contrast, predictions based on the unified national and landmark standards achieve relatively good accuracy, with the national standard showing an overall error of approximately 4%, yet still lower than that of the RF model developed in this study. The proposed RF model exhibits an overall error of about 1%, offering the highest accuracy and best stability. Therefore, the RF model provides a more accurate and reliable prediction of component ultimate bearing capacity.

### 5.2. Compared with Other Algorithm Models

To further evaluate algorithm performance, six machine learning models and one deep learning model were applied for data modeling. In addition to the Random Forest model, benchmark algorithms include Decision Tree, Ridge Regression, k-Nearest Neighbors (KNN), AdaBoost, Support Vector Machine (SVM), and Backpropagation (BP) neural network. Data preprocessing, training set partitioning, 5-fold cross-validation, parameter tuning, and hyperparameter optimization were applied consistently across all models.

**Figure 17:**
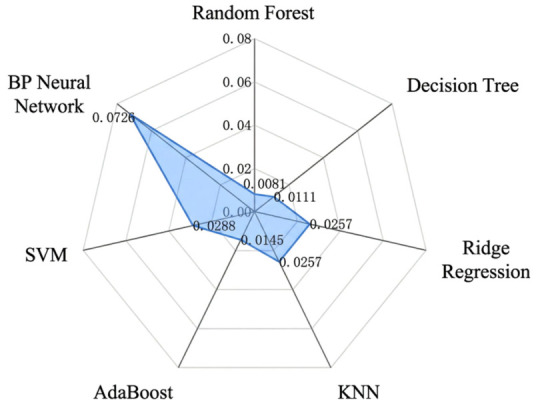
Comparison of different algorithm models MSE for CFST components.

Comparative results are presented in Table 5 and Figure 17. These show that the BP neural network model has the highest index but relatively poor performance. KNN and Ridge Regression exhibit identical indices, reflecting similar predictive ability. The Decision Tree model performs better than these, yet remains inferior to the Random Forest model, which achieves the lowest index of 0.0081, indicating superior performance. Thus, the Random Forest algorithm provides the most accurate prediction of ultimate bearing capacity for CFST components.

**Figure 18:**
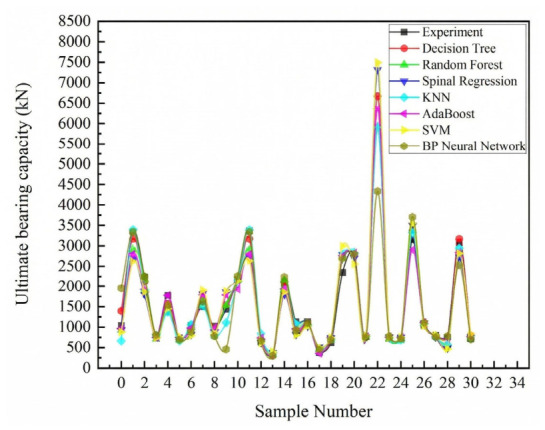
Randomly select samples to predict the effect of each model.

**Figure 19:**
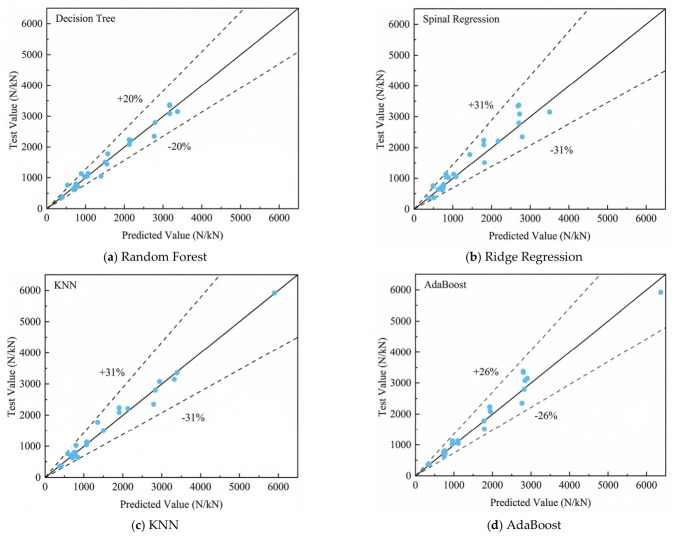

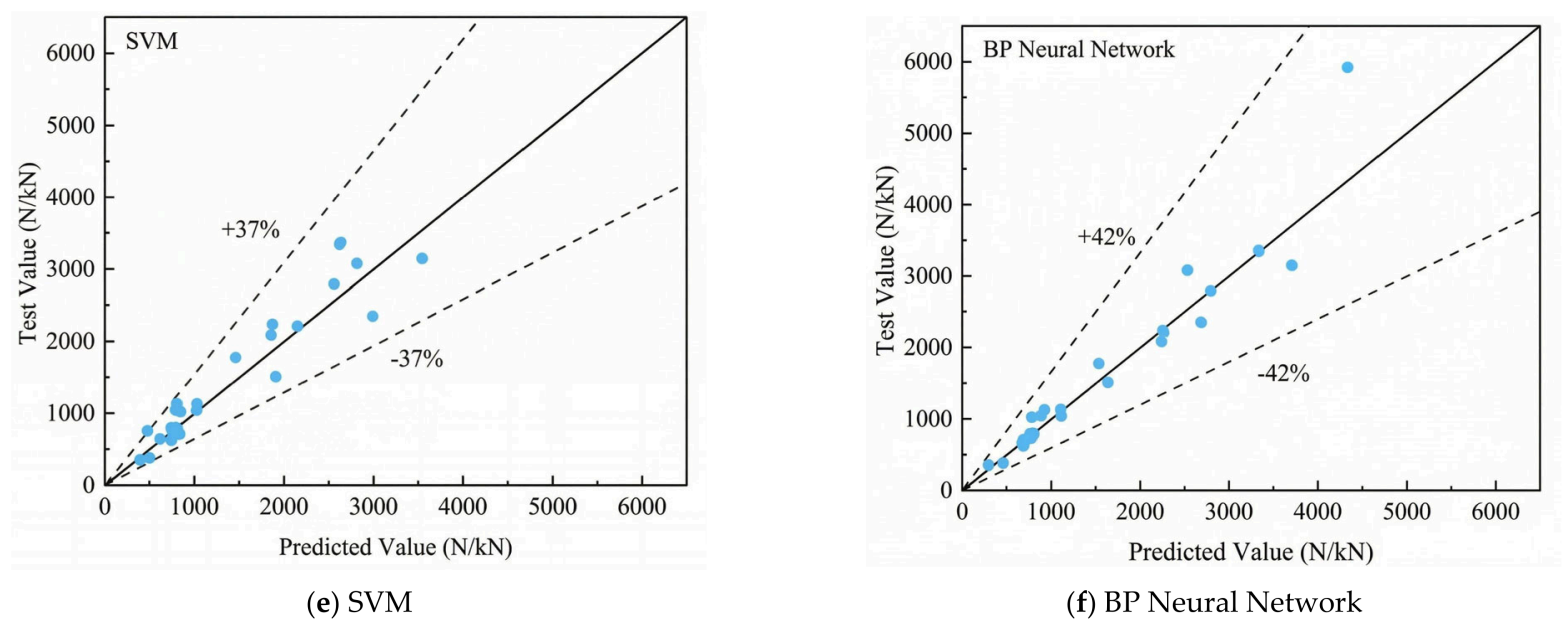
Comparison of prediction and experimental results of other algorithms.

Figure 18 and Figure 19 show that the Decision Tree model achieves good curve fitting, with data points evenly distributed around the isoline and a maximum deviation within 20%, though stability is lower than the Random Forest model. Ridge Regression and KNN have maximum deviations within 31%, with KNN exhibiting better stability. AdaBoost performs slightly worse than the Decision Tree, with a maximum deviation of 26% and moderate stability. SVM and BP neural network models perform the poorest, with maximum deviations of 37% and 42%, and scattered data points. In contrast, the Random Forest model, shown in Figure 14b, produces evenly distributed points largely overlapping the isoline, with a maximum deviation within 15%. Overall, while the proposed model demonstrates significant improvements over existing models, these results should be interpreted with caution. The observed performance gain partly stems from the model tuning processes, and the model’s predictions are currently limited to columns of the heights tested. Further studies are required to generalize these findings to different loading conditions and structural configurations. The improved model predictions closely match experimental results, exhibiting minimal deviation and superior performance.

This study addresses the regression prediction task on structured tabular data (24-dimensional features, 154 samples). In such small-scale structured data scenarios, models such as Random Forest and BP neural network are recognized as mainstream benchmarks in the academic community, whereas CNN and GNN are primarily designed for image or topological data. Forced adaptation not only fails to leverage their structural advantages but may also introduce overfitting risks due to over-parameterization. The input features systematically integrate mechanical mechanism parameters, including section constraint effect, shape coefficient, and composite strength, and are comprehensively compared with the design codes of six countries. This effectively internalizes domain knowledge from traditional physical models into a data-driven framework, establishing an indirect benchmark against physics-informed methods. Furthermore, as demonstrated in Figure 19, extensive experiments involving seven types of models (covering classical regression, ensemble learning, and basic deep networks) confirm the significant advantages of Random Forest in prediction accuracy and robustness, which remain sufficiently stable within the current comparative framework.

## 6. Experimental Verification

### 6.1. Experimental Design and Discussion

To validate the accuracy of the Random Forest prediction model for ultimate bearing capacity under uniaxial compression, nine concrete-filled steel tubular column specimens were tested using a YAW-10000J microcomputer-controlled electro-hydraulic servo compression-shear machine (Beijing Times peak Technology Co., Ltd., Beijing, China). The measured ultimate capacities were compared with the predictions from the RF model.

#### 6.1.1. Mechanical Property Indicators of Steel Tubes

The steel material property test used samples from the same batch of steel as the steel tubes, with three specimens (A1–A3). The steel tubes were processed into standard test specimens, and their basic mechanical properties were measured using the metal tensile testing method. The test was displacement-controlled, with a loading speed of 1 mm/min [53]. The shape and dimensions of the test specimens followed the standard specifications.

The steel tube material properties were tested using a microcomputer-controlled electronic universal testing machine (Changchun new testing machine Co., Ltd., Changchun, China). The force control system employs a fully digital AC servo controller, while deformation is measured via the relative displacement of the upper and lower clamps, processed through frequency doubling and shaping by digital circuits.

The average test results for the yield limit *f_y_*, ultimate strength *f_u_*, and elastic modulus *E_s_* of each specimen material are presented in Table 6.

#### 6.1.2. Mechanical Performance Indicators of Concrete

The concrete strengths were divided into two types: C50 and C30, using Conch PO42.5 cement and PO32.5 cement, respectively. The sand used was medium-coarse sand, and the stone particle size ranged from 5 to 35 mm. The mix proportion for C50 concrete (cement/sand/stone/water) was 1:1.04:2.21:0.34, while the mix proportion for C30 concrete (cement/sand/stone/water) was 1:1.24:2.85:0.45, with the addition of high-strength water reducer FDN at 1% of the cement content. For each grade of concrete, two sets of 150 × 150 × 150 mm^3^ concrete cube test blocks were reserved and cured under the same conditions as the test specimens, for measuring the compressive strength (*f_cu_*) of the concrete. After the concrete test blocks reached the specified age, uniaxial compressive tests were conducted using the YZ200A pressure testing machine (Hongshan testing machine factory, Wuhan, China), following the test method specified in GB/T50081-2002 [54]. It is worth noting that while standard uniaxial tests suffice for CFST components, the mechanical responses of larger-scale concrete structures involve more complex nonlinear coupling and dynamic interactions, such as the seismic fluid–structure interactions in concrete gravity dams [55] and the dynamic deformation monitoring in high-rise structures [56]. Each set of test blocks consisted of 3 samples (with their average values taken), for a total of 4 sets and 12 test blocks. The test block numbering followed the example of C30-07-01, where C30 indicates the concrete strength, 07 indicates the test pressure at 7 days, and 01 indicates the number of the test block in that set. The test results of each test block were comprehensively considered during the calculation and analysis process. The material property test results of the concrete are shown in Table 7.

#### 6.1.3. Specimen Size Parameters

**Figure 20:**
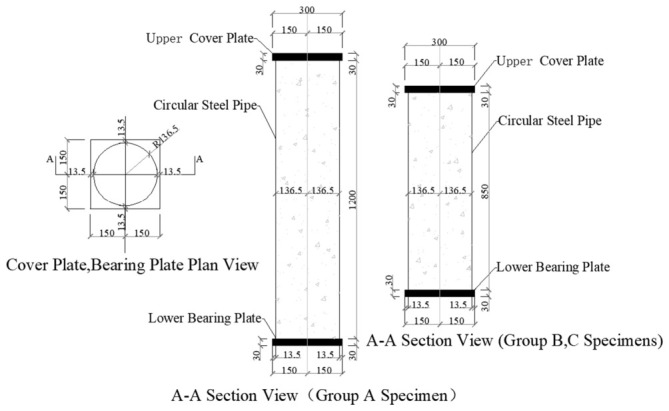
The dimension drawings of specimen (unit: mm).

The experiment used steel tubes with uniform cross-sectional dimensions of *Φ*273 × 6 mm, an outer diameter of 273 mm, a wall thickness of 6 mm, and material grade Q235B. The steel tube components were spiral tubes. Parameters for each test specimen are listed in Table 8. The test piece should be processed strictly according to the dimensions shown in Figure 20.

#### 6.1.4. Specimen Fabrication and Loading

During specimen casting, concrete was densely vibrated and compacted every 30 cm using a *Φ*50 immersion vibrator. At mid-height, a self-fabricated epoxy-coated steel mesh frame was embedded into the column. Pouring continued for the remaining concrete, compacted by manual vibration and uniform tapping of the steel tube, followed by surface finishing. Specimens were cured at room temperature with regular watering for 28 days.

#### 6.1.5. Test Piece Testing Process

**Figure 21:**
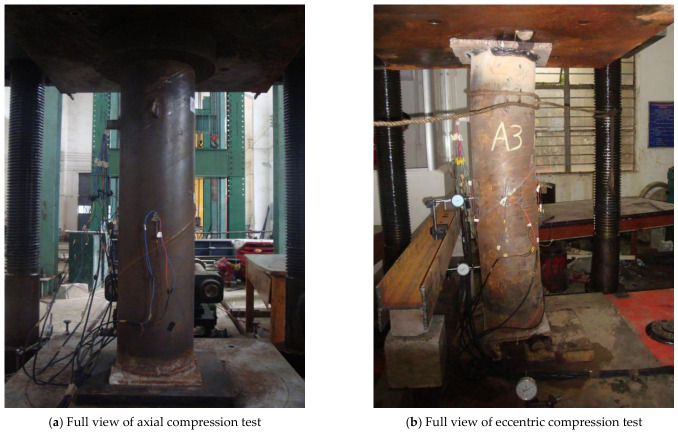
Test operation condition.

As shown in Figure 21, the YAW-10000J microcomputer-controlled electro-hydraulic servo testing machine applies load to the specimen. Before the eccentric compression test, two Φ8 steel bars were welded on the upper and lower pads at the designated eccentric distances to form a groove with the base plate, preventing roller shaft slippage during installation and loading. A triangular block was placed under the side opposite the eccentric force to stabilize the specimen, and removed slowly after loading. Loading was terminated, and data was recorded when the specimen’s bearing capacity fell below 80% of the peak load or severe deformation occurred.

#### 6.1.6. Ultimate Bearing Capacity of Test Specimen

**Figure 22:**
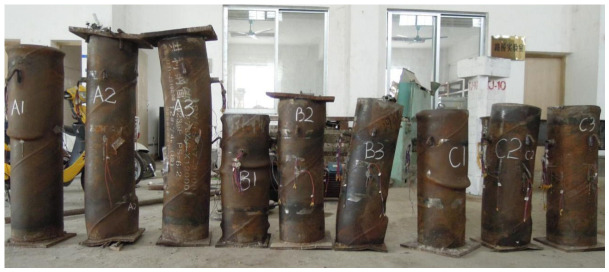
Failure mode of all specimens.

The failure modes of all test specimens are shown in Figure 22. Axial compression specimens (A1, B1, and C1) exhibit similar behavior, demonstrating good ductility and post-peak load-bearing capacity. During the elastic phase, no notable changes occur. At approximately 80% of peak load, cross-diagonal cracks form on the outer steel tube wall. These cracks propagate, with rust layers peeling off, indicating yielding. As the load increases, buckling occurs at vulnerable locations such as edges, weld ends, or openings with stress concentration, accompanied by a sharp increase in circumferential strain and intensified confinement. Upon failure, significant bulging appears at the ends and midsection of the specimen, with diagonal shear slip.

All eccentric compression specimens exhibited slow increases in strain and deflection during initial loading, with no significant deformation. As vertical load approached the ultimate capacity, strain and deflection increased rapidly, with midpoint displacement exceeding that at the quarter points, indicating pronounced mid-span flexural deformation. During the descending or plateau phase, steel tubes in the top and bottom compression zones buckled, showing evident bulging. Maximum lateral displacement occurred at the specimen midsection. The transverse deformation profile resembled a half-wave sine curve, with a concave center and outward bulging at the ends. Upon failure, steel tube rupture and concrete cracking were audible, and most strain gauge readings overflowed, terminating the test. The test results of the ultimate bearing capacity of each component are shown in Table 9.

### 6.2. Model Validation

**Figure 23:**
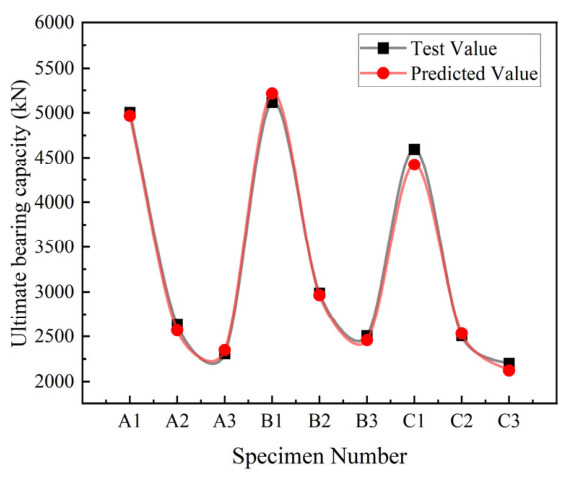
Verification of prediction results of RF model test for CFST members.

This section analyzes and verifies the predictive effectiveness of the RF model for ultimate bearing capacity using CFST component loading test data. As shown in Table 10 and Figure 23, the curve of ultimate bearing capacity predicted by the Random Forest model closely aligns with experimental values, indicating minimal deviation and high accuracy. The RF model effectively captures the mechanical behavior of CFST components under various conditions, providing accurate and reliable predictions.

**Figure 24:**
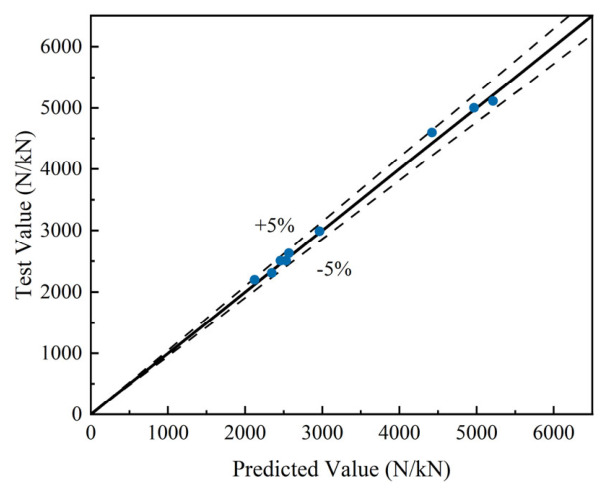
Comparison of ultimate bearing capacity between five prediction models and those measured.

Using the Random Forest algorithm model, we obtained predicted values for the ultimate bearing capacity of components. The mean (*AVG*) and coefficient of variation (*δ*) of the ratio between predicted (*N*) and experimental (*N_t_*) values were calculated, as shown in Figure 24 and Table 10. The predicted data points are nearly evenly distributed, indicating minimal error between predicted and experimental values. The data points are symmetrically distributed around the isoline, with most coinciding with it. The maximum deviation is within 5%, representing a 10% improvement in prediction accuracy over previous models. It should be noted that the model was primarily trained for axial compression loads, and its accuracy in eccentric loading scenarios may be limited and requires further validation. However, this still confirms the effectiveness of the model’s experimental prediction verification. The predicted ultimate bearing capacity closely matches experimental values, demonstrating excellent training results. Thus, the Random Forest algorithm model is both feasible and efficient for predicting the ultimate bearing capacity of CFST components in this paper.

Comparison of predicted ultimate bearing capacity from the RF model shows that the mean of the test validation model is 0.992, a 0.3% improvement over the predicted model mean of 0.989. The test validation model exhibits a mean square deviation of 0.017 and a coefficient of variation of 0.017, compared to 0.093 and 0.094 for the predicted model, representing reductions of 81.7% and 81.9%, respectively. These results confirm the model’s prediction stability, with reduced deviation variance indicating excellent performance.

### 6.3. Uncertainty Quantification and Sensitivity Analysis

In this study, the uncertainty quantification and sensitivity are quantified by the statistical distribution of the ratio of the predicted value to the experimental value. As shown in Table 3 and Figure 15, the *AVG* of the training set is 1.002 and the test set is 0.989, both of which are close to 1, indicating that the overall prediction deviation is very small; *σ* and *COV* of the training set were 0.058 and 0.058, respectively, while those of the test set were 0.093 and 0.094, reflecting the low degree of dispersion of the prediction results. From the scatter distribution in Figure 13b and Figure 14b, the data points are closely clustered around the isoline, and the maximum deviation of training and testing is controlled within 15%, which further confirms the good robustness of the model. The comparative analysis with national design specifications and other machine learning algorithms (Table 4 and Table 5) shows that the *COV* (0.094) of the Random Forest model in this paper is significantly lower than that of the current specifications (0.178–0.223) and other methods, showing the lowest predictive variability and optimal stability. In addition, based on the independent test verification of nine CFST columns, the average between prediction and test ratio is 0.992, *σ* and *COV* are only 0.017, and the maximum error is within 5%, indicating that the model has high reliability in the actual bearing capacity inference, and its prediction uncertainty is at a very low level within the acceptable range of the project.

## 7. Conclusions

Through research on concrete-filled steel tubes, based on experimental data and combining existing experimental data, a prediction model for the ultimate bearing capacity of concrete-filled steel tubes was established using the RF algorithm. Experimental verification was conducted to assess the accuracy of the model, and the prediction results were evaluated based on the *MSE* index. The following conclusions were drawn:

(1) The steel tube section moment of inertia (*I_s_*) contributes the most to the bearing capacity, accounting for 35.56%; other significant factors include steel tube cross-sectional area (*A_s_*, 17.25%), component diameter (*D*, 8.74%), concrete section moment of inertia (*I_c_*, 8.33%), column length (*L*, 5.01%), concrete area (*A_c_*, 4.78%), and steel tube wall thickness (*t*, 3.31%), highlighting their critical importance in design considerations.
(2) Compared with other algorithm models, the average accuracy of the improved RF model is increased by 42%. The average prediction and experimental ratios of the training set and the test set are 1.002 and 0.989, respectively. The maximum deviation is within 15%, and the mean square error is 0.0081. The improved RF model is superior to other algorithms in prediction accuracy, robustness and data fitting. Compared with the design specification, the overall error is about 1%, which proves its superior reliability in accurately and consistently predicting the ultimate bearing capacity of concrete-filled steel tubular members.
(3) Specialized experiments further validated the accuracy and robustness of the RF model. For nine CFST column specimens varying in concrete strength, column length, slenderness ratio, and eccentricity, the maximum deviation between measured ultimate bearing capacity and RF predictions was below 5%. Moreover, predicted and experimental curves showed excellent agreement, confirming that the RF model can accurately predict the bearing capacity of CFST members under both axial and eccentric compression across a wide range of parameters.

Looking ahead, translating the proposed RF model into a practical solution for structural engineers represents a promising avenue, which we plan to actively pursue in our subsequent work. Given the model’s high accuracy and computational efficiency, exploring its deployment as a cloud-based API can be pursued to enable seamless integration into Building Information Modeling (BIM) software (Revit 2025). This will allow real-time predictions to be obtained directly within design workflows. Furthermore, developing interactive web applications can be explored to democratize access to this data-driven tool without requiring machine learning expertise. Beyond CFST structures, extending the proposed methodology—integrating comprehensive feature engineering, ensemble learning, and mechanics-interpretable feature importance—to other concrete structures characterized by nonlinear behavior and complex interactions can also be investigated. For instance, in concrete gravity dams and high-rise structures, where complex coupling effects critically affect structural integrity, applying this interpretable RF framework can be explored not only to predict structural responses but also to identify dominant influencing factors, thereby practically advancing intelligent structural design. The current stochastic forest models give deterministic prediction values, which fail to quantify the uncertainty of the prediction itself. For structural reliability analysis or probability-based design, if the prediction results lack a confidence interval or distribution information, the uncertainty quantification method can be introduced on the basis of the existing model in the subsequent work. For example, the bootstrap mechanism of Random Forest or quantile regression forest can be used to directly output the confidence interval or probability distribution of the predicted value. We can also try to link this kind of prediction with the structural reliability calculation method to estimate the bearing capacity failure probability or reliability index, so as to better serve the probability-based assessment and design.

## Figures and Tables

**Table 1:** Data descriptive statistics table.

Feature Names	Count	Mean	Std	Min	25%	50%	75%	Max
*D*	154	140.97	42.93	82.60	112.56	129.00	165.00	320.00
*t*	154	3.68	2.11	1.00	2.89	3.00	4.65	12.00
*L*	154	411.96	104.39	200.00	340.00	406.50	500.00	660.00
*f_y_*	154	339.35	57.2	222.70	303.50	338.90	360.00	492.80
*f_cu_*	154	66.81	26.23	9.60	47.82	58.50	84.40	139.30
*fc*	154	57.60	25.12	7.70	38.23	48.50	74.40	129.30
*fck*	154	48.11	21.61	4.93	32.06	40.57	62.22	111.68
*λ*	154	11.93	1.80	3.30	12	12.10	12.60	14.00
*A_c_* (×10^3^)	154	15.40	11.80	5.00	8.96	11.94	19.11	73.54
*A_s_*	154	1640.84	1225.38	279.60	995.72	1334.06	1894.41	6883.23
*I_c_* (×10^6^)	154	29.90	68.79	19.91	63.82	11.35	29.07	43.04
*I_s_* (×10^3^)	154	5961.86	13,303.99	276.88	1498.04	2643.88	5273.98	84,335.04
*E_c_* (×10^3^)	154	37.64	5.10	23.77	34.10	36.45	41.33	49.01
*E_s_* (×10^3^)	154	205.76	1.16	200.00	206.00	206.00	206.00	206.00
*ξ*	154	1.54	4.15	0.09	0.47	0.83	1.27	33.21
*B*	154	1.23	0.04	1.14	1.20	1.23	1.24	1.34
*C*	154	−0.22	0.11	−0.55	−0.29	−0.18	−0.14	0.01
*f_scg_*	154	101.35	38.22	29.11	71.21	90.12	125.86	230.30
*f_scd_*	154	97.81	39.49	27.15	66.91	87.03	122.57	211.70
*C*_1_	154	7.54	0.29	7.21	7.44	7.51	7.53	8.96
*C*_2_	154	0.79	0	0.77	0.79	0.79	0.79	0.79
*f_cc_*	154	137.66	51.88	42.46	101.27	119.45	171.09	280.51
*f_yr_*	154	267.71	45.62	175.79	239.40	267.32	284.04	388.73
*f_ym_*	154	909.28	483.55	305.94	634.65	806.53	971.06	2899.32
*N_t_*	154	1711.79	1160.66	341.90	785.75	1495.50	2327.50	7914.00

Note: *D* represents the diameter of the concrete-filled steel tube; *t* denotes the wall thickness of the component steel tube; *L* signifies the length of the component column; *f_y_* stands for the yield strength of steel; *f_cu_* indicates the compressive strength of concrete cubes; *f_c_* signifies the compressive strength of concrete cylinders; *f_ck_* represents the standard value of axial compressive strength of concrete; *λ* is the slenderness ratio of the component; *A_c_* is the area of concrete; *A_s_* is the area of steel tube; *I_c_* is the moment of inertia of the concrete section; *I_s_* is the moment of inertia of the steel tube section; *E_c_* is the elastic modulus of concrete; *E_s_* is the elastic modulus of steel; *ξ* is the confinement coefficient of the component; *B* and *C* are the influence coefficients of section shape; *f_scg_* is the combined compressive strength considering the influence coefficient of section shape; *f_scd_* is the combined compressive strength; C_1_ and *C*_2_ are calculation parameters related to the aspect ratio; *f_cc_* is the enhanced characteristic strength of concrete under axial load; *f_yr_* is the yield strength of steel considering the influence of *C*_2_; *f_ym_* is the yield strength of steel considering the influence coefficient of concrete inside the tube; *N_t_* is the ultimate bearing capacity of the component; the reference values for *C*_1_ and *C*_2_ related to the aspect ratio are as follows: when *L*/*D* equals 0, *C*_1_ is taken as 9.47 and *C*_2_ is taken as 0.76; when *L/D* equals 5, *C*_1_ is taken as 6.40 and *C*_2_ is taken as 0.80; when *L/D* equals 10, *C*_1_ is taken as 3.81 and *C*_2_ is taken as 0.85; when *L/D* equals 15, *C*_1_ is taken as 1.80 and *C*_2_ is taken as 0.9; when *L/D* equals 20, *C*_1_ is taken as 0.48 and *C*_2_ is taken as 0.95; and when *L/D* equals 25, *C*_1_ is taken as 0 and *C*_2_ is taken as 1.0.

**Table 2:** Test data of axial compression.

Specimen Number	Predicted Value (kN)	Experimental Value (kN)	Specimen Number	Predicted Value (kN)	Experimental Value (kN)
1	1040	947	17	1140	1108
2	3370	2898	18	381.4	399
3	1930.6	2176	19	635	728
4	745	714	20	2354	2777
5	1773.8	1534	21	2746	2832
6	686	695	22	747	778
7	1058	1015	23	5904	5927
8	1500	1637	24	735	738
9	1024	944	25	730	700
10	1440	1540	26	3150	3444
11	2273	2171	27	1090	1108
12	3364	2898	28	770	775
13	680	746	29	500	624
14	346.5	348	30	3099.3	2857
15	2077.6	2176	31	757	760
16	1117	1015			

**Table 3:** Comparison of different codes.

	Training Set	Test Set
*AVG*	1.002	0.989
*σ*	0.058	0.093
*COV*	0.058	0.094

**Table 4:** Results of different codes.

Statistics	GB 50936-2014	DBJ/T13-51-2010	AISC	EC4	AIJ	BS5400	This Paper’s RF Mode
*AVG*	0.962	0.955	0.844	1.017	0.887	0.805	0.989
*COV*	0.186	0.223	0.179	0.182	0.178	0.199	0.094

**Table 5:** Statistical table of regression model test set indicators.

Model Name	Decision Tree	Random Forest	Ridge Regression	KNN	AdaBoost	SVM	BP Neural Network
*MSE*	0.01110	0.00810	0.02571	0.02571	0.01447	0.02880	0.07264

**Table 6:** The materials mechanics performance of steel tube.

Specimen Number	Specimen Thickness (mm)	Average Yield Strength *f_y_* (N/mm^2^)	Average Yield Strain (*ε_AVG_*)	Ultimate Strength *f_u_* (N/mm^2^)	Elastic Modulus *E_s_* (N/mm^2^)
A1–A3	3.8	325	1578	450	2.19 × 10^5^

**Table 7:** The materials mechanics performance of concrete test.

Concrete Type	Specimen Size (mm)	Specimen Number	Compressive Strength (MPa)	Average 7-Day Strength (MPa)	Average 28-Day Strength (MPa)
C30	150 × 150 × 150	C30-07-01	30.67	29.2	-
		C30-07-02	28.00		
		C30-07-03	28.89		
		C30-28-01	36.80	-	36.8
		C30-28-02	35.51		
		C30-28-03	38.09		
C50	150 × 150 × 150	C50-07-01	46.67	44.7	-
		C50-07-02	42.67		
		C50-07-03	44.89		
		C50-28-01	54.65	-	58.8
		C50-28-02	59.32		
		C50-28-03	62.43		

**Table 8:** Number and main parameters of specimens.

Specimen Number	Steel Tube Dimensions *D × t × L* (mm)	Concrete Strength	Confinement Coefficient *ξ*	Slenderness Ratio *λ*	Eccentricity *e* (mm)	Eccentricity Ratio *e/r*_0_
A1	273 × 6 × 1200	C50	0.957	17.582	0	0
A2			0.957	17.582	40	0.3065
A3			0.957	17.582	80	0.6130
B1	273 × 6 × 850	C50	0.957	12.454	0	0
B2			0.957	12.454	40	0.3065
B3			0.957	12.454	80	0.6130
C1	273 × 6 × 850	C30	1.546	12.454	0	0
C2			1.546	12.454	40	0.3065
C3			1.546	12.454	80	0.6130

**Table 9:** Test results of the specimens.

Specimen Number	Concrete Strength	Column Length (mm)/Slenderness Ratio	Eccentricity (mm)	Ultimate Bearing Capacity (kN)	Displacement at Ultimate Load (mm)	Moment at Ultimate Load (kN·m)
A1	C50	1200/17.6	0	5020	10.3	—
A2			40	2614	2.7	111.6
A3			80	2296	4.1	193.1
B1	C50	850/12.4	0	5110	6.7	—
B2			40	2980	4.3	132.0
B3			80	2492	5.5	213.1
C1	C30	850/12.4	0	4589	7.8	—
C2			40	2504	4.0	110.2
C3			80	2161	8.0	190.2

**Table 10:** Test data of axial compression for CFST.

Specimen Number	A1	A2	A3	B1	B2	B3	C1	C2	C3
Predicted value	4969.8	2561.72	2318.96	5212.2	2950.2	2442.16	4451.33	2529.04	2117.78
Test value	5020	2614	2296	5110	2980	2492	4589	2504	2161

## Data Availability

The original contributions presented in this study are included in the article. Further inquiries can be directed to the corresponding author.
